# Evaluation of a Collaborative Care Program for Patients With Treatment-Resistant Schizophrenia: Protocol for a Multiple Case Study

**DOI:** 10.2196/35336

**Published:** 2022-06-13

**Authors:** Amy Jongkind, Michelle Hendriks, Koen Grootens, Aartjan T F Beekman, Berno van Meijel

**Affiliations:** 1 Reinier van Arkel ’s-Hertogenbosch Netherlands; 2 Amsterdam University Medical Center (VUmc) Department of Psychiatry Amsterdam Public Health Research Institute Amsterdam Netherlands; 3 Tranzo, Tilburg School of Social and Behavioral Sciences Tilburg University Tilburg Netherlands; 4 Inholland University of Applied Sciences Department of Health, Sports & Welfare Cluster Nursing Amsterdam Netherlands; 5 Parnassia Psychiatric Institute Parnassia Academy The Hague Netherlands

**Keywords:** treatment-resistant schizophrenia, collaborative care, recovery, personalized care, clozapine, lifestyle, peer support, shared decision-making, motivational interviewing, nurse-led intervention

## Abstract

**Background:**

Approximately one-third of all patients with schizophrenia are treatment resistant. Worldwide, undertreatment with clozapine and other effective treatment options exist for people with treatment-resistant schizophrenia (TRS). In this respect, it appears that regular health care models do not optimally fit this patient group. The Collaborative Care (CC) model has proven to be effective for patients with severe mental illness, both in primary care and in specialized mental health care facilities. The key principles of the CC model are that both patients and informal caregivers are part of the treatment team, that a structured treatment plan is put in place with planned evaluations by the team, and that the treatment approach is multidisciplinary in nature and uses evidence-based interventions. We developed a tailored CC program for patients with TRS.

**Objective:**

In this paper, we provide an overview of the research design for a potential study that seeks to gain insight into both the process of implementation and the preliminary effects of the CC program for patients with TRS. Moreover, we aim to gain insight into the experiences of professionals, patients, and informal caregivers with the program.

**Methods:**

This study will be underpinned by a multiple case study design (N=20) that uses a mixed methods approach. These case studies will focus on an Early Psychosis Intervention Team and 2 Flexible Assertive Community treatment teams in the Netherlands. Data will be collected from patient records as well as through questionnaires, individual interviews, and focus groups. Patient recruitment commenced from October 2020.

**Results:**

Recruitment of participants commenced from October 2020, with the aim of enrolling 20 patients over 2 years. Data collection will be completed by the end of 2023, and the results will be published once all data are available for reporting.

**Conclusions:**

The research design, framed within the process of developing and testing innovative interventions, is discussed in line with the aims of the study. The limitations in clinical practice and specific consequences of this study are explained.

**International Registered Report Identifier (IRRID):**

DERR1-10.2196/35336

## Introduction

### Background

Approximately 20% to 30% of patients with schizophrenia appear to be resistant to treatment. This means that current antipsychotics insufficiently affect this patient group, despite adequate dosage and duration of treatment with antipsychotics [1]. Compared with patients with schizophrenia who are not treatment resistant, those with treatment-resistant schizophrenia (TRS) display more social, functional, and economic problems [2] and poorer cognitive functioning, in addition to being at a higher risk of metabolic syndrome and other health-related problems due to their unhealthy lifestyle [3]. For example, these patients consume more drugs, nicotine, and alcohol [2]. Suicidal behavior is more prevalent [2], and it also causes a higher burden of disease [4] than patients who are not treatment resistant. Finally, studies have also shown that treatment adherence often proves challenging owing to cognitive problems, low motivation, side effects of sedative use, and psychiatric symptoms such as hallucinations and delusions [5].

Despite the denotation *treatment resistant*, there is still an evidence-based treatment option available for this patient group, namely clozapine treatment or optimizing treatment with clozapine if patients are already using it. Several treatment options are available for clozapine resistance, such as pharmacological additions to clozapine, physical exercise, cognitive behavioral therapy, music therapy, and electroconvulsive therapy [1,6,7].

Resistance can be considered as incomplete recovery [8]. Within clinical practice, the mentioned treatment options are often not optimally offered [1]. For example, the suboptimal prescription of clozapine constitutes a serious problem in the treatment of patients with TRS, because both prior use of clozapine and fewer pre–clozapine antipsychotic trials have been associated with better treatment outcomes for people with TRS [9]. The effectiveness of clozapine is superior and unique to TRS [10]. Clinical practice guidelines are typically not followed by prescribers, and, as such, clozapine remains underused in patients [11]. More than half of all individuals with schizophrenia receive either no treatment or suboptimal treatment [11], whereas approximately 95% of people with schizophrenia do not receive an appropriate combination of evidence-based services [12].

Evidently, there is a notable gap between the available treatment options and their uptake in daily practice. In some cases, care professionals simply feel that patients should not be burdened by further treatment after having previously undergone unsuccessful treatment. Demoralization among both patients and professionals can occur, thus leading to both sides having little hope for improvement [13]. Psychiatrists are often reluctant to prescribe clozapine, in part, owing to problems in regulating its side effects, which often leads patients to discontinue clozapine. Other reasons for underprescribing clozapine include the inconvenience of therapeutic blood monitoring [6], lack of knowledge and training regarding clozapine among care professionals [14], lack of clarity over diagnosis, difficulty in correctly identifying patients for this treatment, service fragmentation, and lack of adequate training of health care professionals in clozapine use [15]. Another reason for non–guideline-compliant treatment is that patients with TRS either refuse treatment or display poor adherence [16].

This paper introduces the Collaborative Care (CC) model as a means to improve the quality of care among patients with TRS. CC has proven to be effective for other serious mental illnesses, such as depressive, bipolar, and personality disorders [17-20]. The CC model is predicated on the principles that both patients and their informal caregivers should form part of the treatment team, that a structured personalized treatment plan should be put in place with planned evaluations by the team, and that the approach should be multidisciplinary in nature and make use of evidence-based interventions. In CC, the patient is in a position to manage their own treatment. Within the team, a personalized treatment plan is set up along with clear recovery goals that will be evaluated every 3 months. Important treatment decisions are jointly made within the team based on shared decision-making (SDM) principles. In CC, medical, psychological, and social interventions are integrated, which means that it is a holistic approach that incorporates treatments that have been shown to be effective on their own, within a coherent longitudinal treatment framework.

Given that a CC program for patients with TRS (CC-TRS) was not yet available, we developed such a program ourselves. Previous research provides evidence of the value of treatment programs that combine medications with a range of psychosocial services in the treatment of people with schizophrenia [21]. The evidence-based interventions provided in the CC-TRS comprise optimal medical treatment (including the use of clozapine), lifestyle interventions, peer support, SDM, and motivational interviewing for informal caregivers. CC-TRS is consistent with the idea that a combination of psychological and psychosocial care with medication treatment is the key factor in maximizing the effectiveness of the treatment of patients with TRS [8,22] and is not only about treating positive psychotic symptoms and responses to antipsychotic medications. We expect that this program will improve quality of care and promote patients’ symptomatic recovery, psychosocial functioning, and quality of life.

However, the implementation of scientific knowledge in daily practice is problematic worldwide. Indeed, there remains a gap in how implementation strategies might improve patient outcomes and health services [23]. Moreover, clozapine use varies widely across settings and countries, whereas patient factors remain relatively consistent. Therefore, not to mention the fact that patients who use clozapine report that its advantages outweigh its disadvantages, it appears that the impact of patient factors is smaller compared with both organizational and clinician-related factors [24].

In this paper, we delineate the research design of a study that aims to gain insight into both the implementation process and preliminary effects of CC-TRS as well as professionals, patients, and informal caregivers’ experiences with the program.

### Intervention: Principles of the CC-TRS

**Figure 1:**
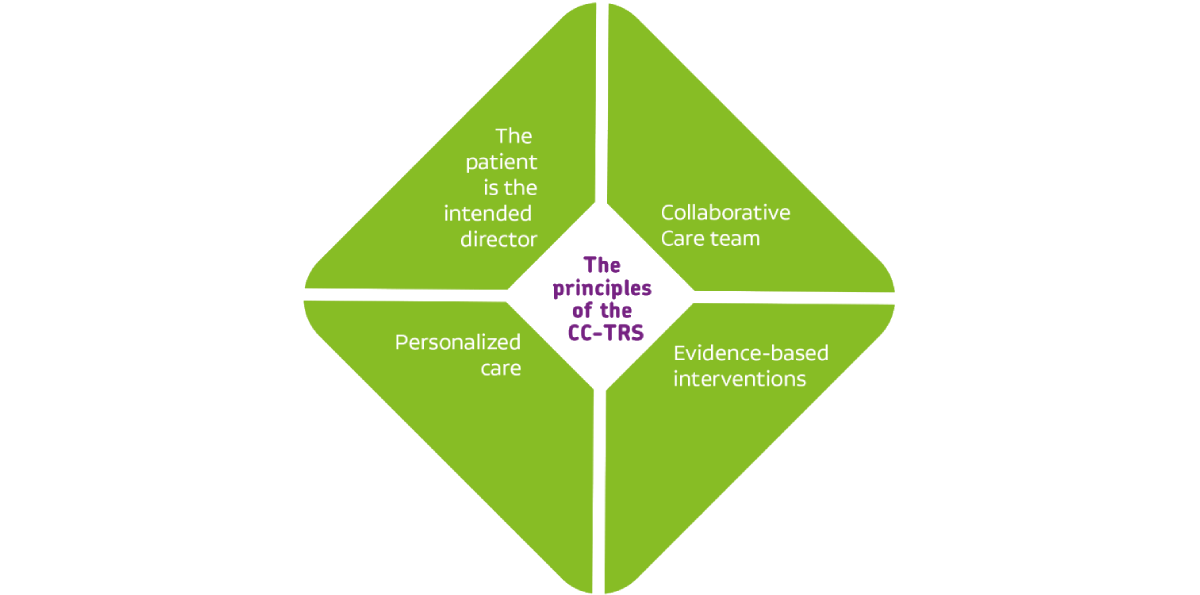
Key principles of the Collaborative Care program for patients with treatment-resistant schizophrenia (CC-TRS).

**Figure 2:**
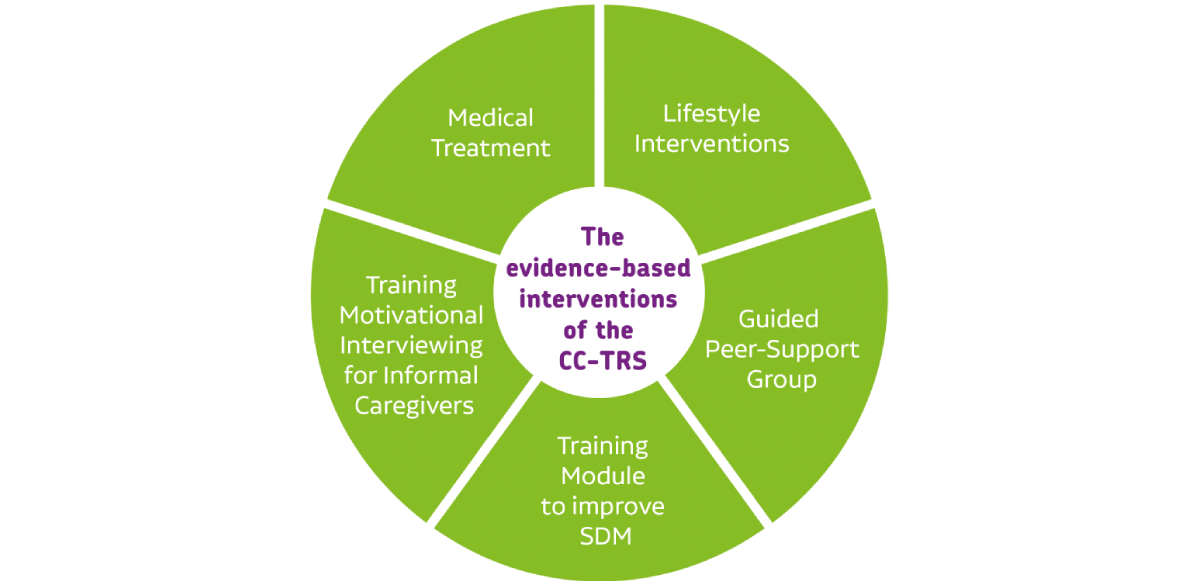
Evidence-based interventions for the Collaborative Care program for patients with treatment-resistant schizophrenia (CC-TRS). SDM: shared decision-making.

There are four key principles that guide the CC-TRS intervention program (Figure 1):

1. To optimize both the continuity and coordination of care, intensive collaboration will be established in a CC team. The CC team will comprise the patient, informal caregiver or caregivers, a care manager, a mental health nurse who will administer the CC-TRS interventions (CC nurse), and a clinical nurse specialist (CNS) who will be responsible for the overall treatment. At all times, the CNS will consult a psychiatrist. There will also be an option to expand the CC team to include other health care professionals to effectively execute the treatment plan.
2. The patient is the intended manager of their own treatment. Other members of the CC team (including informal caregivers) will assist the patient in strengthening their self-management skills and empowerment. Considerations and decisions regarding treatment will be made in collaboration with the CC team according to the principles of SDM [25]. If the patients are not able to manage their own treatment, then other members of the CC team will (temporarily) take over.
3. A personalized treatment plan with clear recovery goals will be developed within the CC team and will be evaluated by the CC team every 3 months. To encourage patient engagement, we have created practical documents for patients, which include a format for a treatment plan and a format for preparing the evaluations of the treatment.
4. Evidence-based interventions are integrated into the treatment plan (see the following sections and Figure 2). These interventions have been proven to be effective in reducing psychotic symptoms and improving social functioning and self-management among patients with persistent psychosis. The caregiver will inform both the patient and informal caregivers about effective treatment options for TRS, such as those included in the CC-TRS. Given the principles of personalized care, the precise trajectory that treatment can take varies among patients, which is why no specific intervention is included as a default in the treatment plan. Moreover, the order and pace at which interventions are delivered can vary. In CC-TRS, the caregiver will continue to both hold out hope for recovery and search for possibilities to improve symptoms and functioning.

### Evidence-Based Interventions Within the CC-TRS

#### Medical Treatment

Given that clozapine is the only effective drug for treating psychotic symptoms in patients with TRS [1,9,26-28], patients in the CC-TRS will be comprehensively informed about the added value of clozapine as part of their recovery. Once patients decide to start clozapine, every effort will be made to ensure that the clozapine treatment is successful. For instance, the psychiatrist will assess both the indications and contraindications for clozapine. If the psychiatrist indicates clozapine and the patient is willing to start, the CNS will prescribe and carry out further treatment with clozapine. The CNS is trained to provide protocol-based clozapine treatment and is legally authorized to carry it out. Clozapine treatment under the care of the CNS is as safe as that under the care of a psychiatrist. Previous research has shown that patients who are monitored by a CNS tend to use clozapine for longer than those monitored by a psychiatrist [29]. Treatment by nurses as opposed to physicians also generally leads to longer consultation times, either more or the same amount of information being provided to patients, and higher patient satisfaction with care [30]. This is because the CNS generally has more available time than the psychiatrist to provide psychoeducation, support the use of eHealth, and carry out motivational interventions [31,32]. The assumption here is that this will encourage adherence to medical treatment, while simultaneously preventing or reducing the common side effects of clozapine (eg, excessive weight gain).

The CNS will prepare for treatment with clozapine by performing the necessary pretreatment checks, as delineated in the guidelines of the Dutch Clozapine Collaboration Group [1]. These checks will also be systematically recorded once again through recourse to the checklist from the Dutch Clozapine Collaboration Group.

We developed an eHealth module for clozapine treatment for patients. This eHealth module provides information about the effects and side effects of clozapine, the course of treatment, and information on the safe use of clozapine. It also provides a diary for patients to monitor their own side effects and a form to evaluate the effects of clozapine.

#### Lifestyle Interventions

The program *Traffic Light Method for Somatic Screening and Lifestyle* [33] will be delivered by a CC nurse who will adopt the role of a lifestyle coach. Weight gain is a common side effect of clozapine [34-36], which is perceived very negatively by patients and, indeed, is commonly cited as a reason to either not start using clozapine in the first place or to discontinue its use [37]. Exercise improves positive and negative symptoms in patients with schizophrenia [38,39]. Hence, with the support of the CC nurse, the patient will develop a lifestyle plan consisting of individual lifestyle goals, with particular attention being paid to exercise and nutrition. By applying the principles of motivational interviewing and SDM, the CC nurse will encourage the patient to adopt a healthy lifestyle. Adoption of a healthy lifestyle will also be encouraged by organizing pleasant lifestyle activities for a group of patients, which can help patients live healthier and produce positive effects on social interaction, mood, and stress [40].

#### Guided Peer-Support Group

A guided peer-support group will be delivered to groups of approximately 8 patients and will involve 90-minute biweekly sessions. We will use a peer-support intervention that was investigated in a randomized clinical trial and found to induce positive effects on social support, self-efficacy, quality of life, negative symptoms, and reduction of distress from symptoms [41]. The patient has the option of joining the group for as long as they want. Each session will follow the same structure, namely, discussing daily experiences in pairs and within the group as a whole. The patients themselves will decide what topics they want to discuss in each session. The CC nurses will guide the groups with minimal professional involvement to stimulate peer-to-peer interaction.

#### Training Module to Improve SDM

A special training was developed by the first author (AJ) in collaboration with care professionals and (former) patients to improve patients’ skills in SDM. Although SDM is mainly advocated to preserve patients’ autonomy, it has also been found to lead to better affective-cognitive outcomes, increased satisfaction, less decisional conflict, and, as such, appears expedient for self-reported health-related patient outcomes [42]. The principles of SDM between patients and professionals are central to this training and should enable patients to be more actively involved in their treatment [25]. A group interview was held with (former) patients about their experiences with SDM during treatment and the specific needs that they felt the training should address. A preliminary version of the training was evaluated by (former) patients and trainers. On the basis of their suggestions for improvement, adjustments were made to the training program. The modified version of the training was again provided to (former) patients by the trainers, after which the final version of the training was formalized. The training consists of psychoeducation and various exercises that seek to build patients’ assertiveness skills, such as asking questions, speaking up, and asking for a second opinion. The training will consist of 4 sessions lasting 2 hours each and will be provided by the CC nurse and a peer-support worker.

#### Training Motivational Interviewing for Informal Caregivers

This training is based on *Family Motivational Intervention* (FMI), which is a form of motivational interviewing training for parents of patients with psychotic vulnerabilities and who engage in cannabis use. This training aims to reduce or stop cannabis use among patients by training parents using motivational interviewing techniques. In our version of the training, informal caregivers will learn communication skills to reduce stress and conflicts and motivate patients to engage in behavior change. FMI training leads to behavioral change in patients, an improvement in patients’ quality of life, and a reduction in both the stress and burden placed on informal caregivers [43]. We modified the original version of the FMI to make it applicable to all informal caregivers of patients with psychosis. Most importantly, the modified FMI does not focus on a predetermined behavioral component such as cannabis use but encompasses all health-promoting behaviors. Our modified FMI training will consist of 7 weekly meetings of 3 hours each, which will be provided by 2 CC nurses.

### Implementation Plan and Training

#### Overview

To implement CC-TRS, an implementation plan will be developed for each participating team [44]. This implementation plan will describe the strategies and responsibilities of each team member during the implementation of CC-TRS. This implementation plan will be developed in collaboration with all team members. Quality consultants will then assist the teams in implementing the CC-TRS.

All team members will have to attend a 60-minute meeting in which the principal investigator will inform them about the study, CC-TRS intervention, and implementation plan. The CNS and CC nurse who will deliver evidence-based CC-TRS interventions will take part in a comprehensive 4-day training course. The CNS and CC nurses will also be supervised and supported by the principal investigator during the implementation of the intervention through at least monthly (telephone) meetings.

#### Research Questions

The study will be underpinned by the following research questions:

1. What course did the implementation process of CC-TRS take, and to what extent was the intervention implemented as originally planned?
2. What barriers and facilitators (ie, factors at the patient, caregiver, and organization levels) influenced its implementation?
3. What treatment options did patients receive during the intervention period?
4. What are the experiences of patients, informal caregivers, and care professionals with the CC-TRS?
5. What are the preliminary effects of CC-TRS on psychosocial functioning, symptoms, quality of life, empowerment, recovery, SDM, self-management, and somatic comorbidities? How can these effects be explained by both the implementation process and the experiences of patients, caregivers, and professionals?

#### Objectives

The purpose of our study is to describe the implementation process of the CC-TRS program in clinical practice and to gain insight into the factors that influence the success of the implementation. We also aim to explicate the relationship between the implementation process (also considering the experiences of participants) and the realized effects at the patient level. This will provide preliminary insights into the effects of such an intervention, both in a variety of circumstances and with respect to different patient variables. The aim of this study is not to quantitatively generalize the results of the sample to the general population but rather to conduct an in-depth analysis of the process of implementation and its attendant outcomes. On the basis of the results of this study, we will subsequently seek to further optimize the CC-TRS intervention.

## Methods

### Design

#### Overview

We will use a multiple case study design that uses a mixed methods approach [45]. The multiple case study design is suitable for research that seeks to gain detailed insight into both the implementation process and preliminary effects of an intervention, as well as the relationship between the implementation process and outcomes. Different perspectives are integrated into the study: the perspective of patients, their informal caregivers, and care professionals. Implementation of the intervention in relation to the outcomes will initially be studied at the individual case level (a single-case analysis). Subsequently, the findings of all cases will be aggregated via a cross-case analysis [45]. Participating patients will be followed up for 1 year during the course of their treatment. The inclusion process was initiated in October 2020.

#### Setting

The study will be carried out in a specialized mental health organization in the Netherlands, with the inclusion of 2 Flexible Assertive Community treatment (FACT) teams and an Early Psychosis Intervention Team (EIT). FACT teams are based on the Assertive Community Treatment model that specifically focuses on patients with serious mental illness who are reluctant to keep appointments at a clinic where outreach care is recommended [46]. Alongside these difficult-to-reach patients, FACT teams are also responsible for the treatment of all patients with severe mental illness in a specific region, with the possibility of intensifying treatment if and when patients experience a crisis. A FACT team treats approximately 200 to 250 outpatients. EITs specialize in the treatment of outpatients in the initial years after the onset of psychosis. Although EITs work in the same way as FACT teams, they spend more time on diagnostics and psychoeducation. Their caseloads are generally smaller and their patients are younger. The EIT, which is part of this study, offers treatment to patients who have presented with a first episode of psychosis and are sometimes treated in this team for several years. The patients included in our study from this EIT met the criteria for TRS.

#### Inclusion Criteria

Patients will be included in the study if they meet the inclusion criteria presented in Textbox 1.

To assess patients’ decisional capacity to consent while taking part in our study, the principal investigator will use the *Brief Assessment of Capacity to Consent* [47]. Patients with impaired capacity will be excluded from this study.

If patients consent to participate, an informal caregiver will also be asked to participate in the study, based on the inclusion criteria presented in Textbox 2.

**Inclusion criteria for patients.**

**Inclusion criteria**

- Aged ≥18 years.
- Have a sufficient command of the Dutch language.
- Diagnosed according to the criteria of the Diagnostic and Statistical Manual of Mental Disorders, Fifth Edition, as having schizophrenia, schizophreniform disorder, schizoaffective disorder, another specified schizophrenia spectrum disorder, other psychotic disorder, unspecified schizophrenia spectrum disorder, or other psychotic disorder.
- Was defined as treatment resistant. In our study, we will determine treatment resistance for each symptom domain (positive and negative domains) using scores on the Positive and Negative Syndrome Scale (PANSS) [48]. Following the remission criteria proposed by Andreasen et al [49], symptoms must be (1) at least moderately severe, that is, a PANSS score of 5 on at least two items or a PANSS score of 6 on 1 item or (2) mildly severe, that is, a PANSS score of at least 4 on one of the items P1, P2, P3, N1, N4, N6, G5, and G9. Moreover, the patient must show impaired psychosocial functioning, with a score of <60 on the Social and Occupational Functioning Assessment Scale [50]. Concerning medical treatment, there must have been 2 adequate treatment episodes in the past with different antipsychotic drugs. The duration of this antipsychotic treatment needs to have been for at least six weeks at the therapeutic dosage. We will use the minimum maintenance dose from the Dutch Summary of Product Characteristics from the Rules Governing Medicinal Products in the European Union to determine the therapeutic dosage criterion. We will also determine medication adherence using data from the electronic prescription system in patients’ files and reports. This will enable us to see whether medication is still being held for patients at a pharmacy that they have not yet picked up.

**Inclusion criteria for informal caregivers.**

**Inclusion criteria**

- The informal caregiver is assigned by the patient.
- The informal caregiver is aged ≥18 years.
- The informal caregiver has a sufficient command of the Dutch language.
- The informal caregiver is able and willing to give informed consent.

#### Sample Size

To gain insight into the implementation process of the CC-TRS, the factors influencing this implementation and preliminary effects at the patient level, sufficient variation in patient characteristics, caregiver characteristics, and the context of implementation are needed. Within the context of the 2 FACT teams and an EIT, we believe that it will be sufficient to include 20 patients in our study to achieve the desired variation. If patients decide to stop the CC-TRS interventions, they will be asked to remain in the study purely for the purpose of carrying out a follow-up analysis.

#### Modifications Due to the COVID-19 Pandemic

Owing to the COVID-19 pandemic, face-to-face contact with participants is no longer self-evident. Therefore, we will have to make modifications to the treatment and measurements of the study depending on the applicable COVID-19 guidelines at that juncture. All modifications will, of course, be in accordance with the advice and guidelines of the mental health organization where the study will be conducted as well as the National Institute for Public Health and the Environment in the Netherlands.

Treatment-based contact will be face-to-face as much as possible. When face-to-face contact is not possible or the patient feels uncomfortable with face-to-face contact and refuses to do so, video calling will be the preferred form of contact, followed by telephone calling.

Team members will preferably engage in contact through video calling. This also applies to feedback meetings with all team members. Measurements with patients will preferably take place face-to-face. Interviews will be conducted via video calling when face-to-face contact is not possible. We will adhere to the recommendations of Opler [51] for conducting Positive and Negative Syndrome Scale interviews via video calling. These recommendations relate to—among other things—video connectivity, high-bandwidth connections, and the webcam's field of view. Research assistants will also receive additional training on how to conduct interviews via video calling.

Group interviews with caregivers and care professionals will take place 1 year after the start of the first inclusion, which means that it will be conducted in October 2022. If there are still restrictions on face-to-face contact at that juncture, group interviews will be conducted by video calling.

#### Recruitment of Patients

Patients who receive treatment in the participating teams will be screened for eligibility for the study. This screening process will divide participants into 3 categories. First, patients who met the inclusion criteria will be invited to participate in the study. Second, patients who do not meet all the inclusion criteria at the time of screening might be potential participants in the future. Third, patients who meet exclusion criteria are excluded. Every 3 months, we will reassess both the patients within the second group and those who are new to the team.

The care manager will screen selected patients supervised by the quality consultants. Patients who meet the inclusion criteria and agree to contact the principal investigator will receive verbal and written information about the study and CC-TRS intervention. The patient will have at least 7 days to consider participation. In the case of consent to participate, the principal investigator will determine the patient’s decisional capacity using the Brief Assessment of Capacity to Consent, over telephone. The patients will be then asked to sign an informed consent form. The reasons for refusal to participate will be registered. The patients will sign an informed consent form before they are included in the study. At T0, we will determine whether a patient is eligible for inclusion in the study.

In the informed consent procedure, patients will be asked to point out an informal caregiver. The informal caregivers will also receive verbal and written information about the study and CC-TRS from the principal investigator. After at least 7 days to consider participation, informal caregivers will be asked for consent for participation in the study. In the case of a positive response, the informal caregiver will sign an informed consent form.

As part of the informed consent procedure, patients will be asked to name an informal caregiver, who will subsequently be asked to give their consent to participate in the study.

#### Research Assistants

Trained research assistants will perform the measurements required for this study. These assistants will not be involved in either the implementation or execution of the CC-TRS. Interrater reliability will be assessed using a simulated interview.

### Data Collection

#### Patients

We will perform document analysis 3 (T3), 6 (T6), 9 (T9), and 12 (T12) months after a patient is included in the study. As part of these document analyses, we will conduct an intervention check to ascertain whether the CC-TRS is being carried out in adherence to the intervention protocol. Therefore, it will be possible to quantify the care received by patients. As part of the intervention, both patients and informal caregivers will discuss their experiences with the CC-TRS as part of their treatment plan evaluations with the CC team. This will be reported in patient files, thus offering the opportunity to gain insight into their experiences with the CC-TRS by conducting document analysis. We expect the CC-TRS to produce positive effects on somatic outcomes such as metabolic syndrome, cardiovascular risks, and (risk of) diabetes mellitus. Therefore, the following somatic outcomes of patients will be extracted from their medical files at baseline (T0) and after 12 months (T12): height, weight, BMI, waist circumference, systolic and diastolic blood pressure, lipid profile, and fasting blood glucose levels. Moreover, the presence of cardiovascular disease, diabetes mellitus, or hypertension will be registered, including the use of medications for these disorders.

There will be 3 face-to-face measurements: when patients are included in the study (T0), after 6 months (T6), and after 12 months (T12). The questionnaires used for quantitative data collection at T0, T6, and T12 are summarized in Table 1. The questionnaires *Consumer Quality Index* and *Experiences with CC-TRS* will only be administered at T12. At T12, after completion of all quantitative measurements, a 60-minute interview will be conducted with the patients. In this interview, a topic list will be used to discuss the results of the quantitative data, the course of the treatment, the implementation of the various treatment components, and the experiences of patients with the CC-TRS. Furthermore, patients will be asked which factors and specific aspects of the CC-TRS influenced the outcomes and whether the desired effects were achieved. All the interviews will be audiotaped and transcribed verbatim.

Patients will receive €7.50 (US $8.02) for each of the first 3 face-to-face measurements and €15 (US $16.04) for the fourth face-to-face measurement in the form of gift vouchers. In addition, their travel expenses will be reimbursed.

**Table 1:** Quantitative measurements of patients at T0, T6, and T12.

Instrument	Description	Validity and reliability
CGI^a^	This is a 3-item scale and the most widely used brief assessment tool in psychiatry for measuring illness severity, global improvement or change, and therapeutic response [52,53].	The psychometric properties of CGI have not yet been established, but clinicians' ratings of psychiatric symptoms correlate significantly with self-rated and other valid scales of symptom severity [54]. Rating scales are an appropriate clinical technique for the measurement of change in antidepressant trials. Global improvement scales would be sufficiently sensitive toto distinguish between responders and nonresponders in clinical [55].
CQi^b^	This is a 15-item questionnaire that allows for the evaluation of outpatient treatment from patients’ perspectives [56].	Research has shown sufficient reliability (Cronbach α between .69 and .95) [57]. The CQi provides valid and reliable results in short-term care for patients in mental health care [58].
DES^c^	This is the Dutch version of the MHRM^d^, a 26-item questionnaire that measures recovery [59].	The Dutch version of the MHRM is a reliable measure (in terms of internal consistency) with a generally acceptable convergent and divergent validity. Cronbach α ranged from .86 to .94 [59].
Dutch PIH^e^	This is a Dutch translation 12-item questionnaire developed in Australia to measure self-management behavior and knowledge among patients with chronic diseases [60].	The PIH exhibits construct validity and internal consistency. Cronbach α is .82 [61]. The PIH has been translated and validated for use among Dutch patients with chronic obstructive pulmonary disease [60].
HoNOS^f^	The HoNOS is a structured interview measuring behavior, impairments, symptoms, and social functioning via the use of 12 items. The instrument has 3 addendums that measure manic symptoms, treatment motivation, and compliance with medication [62].	The Dutch version of the HoNOS has reasonably good psychometric qualities (intraclass correlation coefficient=0.92), can be administrated in a short period, is neither dependent on psychiatric diagnosis nor language, and is regarded as useful by both clinicians and patients [62].
MANSA^g^	This is a 16-item questionnaire that measures the quality of life, with a particular focus on satisfaction with life as a whole and with different life domains [63]. In addition to the MANSA, we will also ask patients to give a score between 0 and 10 to describe their quality of life in general.	The MANSA is a brief instrument for assessing quality of life focusing on satisfaction with life as a whole and with life domains. Its psychometric properties appear satisfactory (Cronbach α=.74 for the satisfaction rating) [63].
PANSS^h^	This is one of the most widely used instruments for measuring the presence and severity of positive, negative, and general psychopathological symptoms of schizophrenia. The PANSS is a 30-item structured interview [48].	The PANSS has good interrater reliability, adequate construct validity, high internal reliability, appropriate test-retest reliability, and external validity [48,64-66].
SDM-Q-9^i^	This is a self-report instrument comprising 9 items that was developed to measure patients’ perceptions of the shared decision-making process (SDM-Q-9) [67].	The SDM-Q-9 has a good acceptance, internal consistency, and acceptable to good convergent validity (Cronbach α=.88) [67].
SOFAS^j^	This is a 1-item rating (0-100) that assesses social and occupational functioning (independently of the severity of psychological symptoms) [50].	To our knowledge, the SOFAS has not yet been tested for psychometric quality. We will use this questionnaire to assess the criteria for treatment-resistant schizophrenia as established by the Working Group of the Treatment Response and Resistance in Psychosis. They use SOFAS to operationalize functional limitations [68].
Questionnaire experiences with treatment	This is a self-developed questionnaire containing 11 items about satisfaction with treatment in general and 4 items about satisfaction with treatment via the Collaborative Care program for patients with treatment-resistant schizophrenia.	The questionnaire has not been examined for psychometric quality.

^a^CGI: Clinical Global Impression.

^b^CQi: Consumer Quality Index (Geestelijke Gezondheidszorg en Verslavingszorg Ambulant).

^c^DES: Dutch Empowerment Scale.

^d^MHRM: Mental Health Recovery Measure.

^e^PIH: Partners in Health scale.

^f^HoNOS: Health of the Nation Outcome Scale.

^g^MANSA: Manchester Short Assessment of Quality of Life.

^h^PANSS: Positive and Negative Syndrome Scale.

^i^SDM-Q-9: Shared Decision-Making Questionnaire 9-item.

^j^SOFAS: Social and Occupational Functioning Assessment Scale.

#### Informal Caregivers

After 12 months (T12), informal caregivers’ experiences regarding both their role in the treatment and the degree of cooperation with the CC team will be measured via a Dutch translation of the *Family Perceptions of Caregiving Role* (FPCR) [69]. The FPCR is a 15-item questionnaire that measures collaboration between informal caregivers and care professionals. To the best of our knowledge, FPCR has not yet been examined for its psychometric quality. However, we consider this questionnaire to be suitable for measuring both the perceived collaboration between informal caregivers and the CC team and the former’s involvement in care. To measure the experiences of informal caregivers with respect to specific elements of CC-TRS, we will use a self-developed 15-item questionnaire called *Evaluation informal caregivers CC-TRS*.

Two 90-minute-long focus group interviews with informal caregivers will be conducted at T12. Given that patients could be enrolled at different points of the study, we plan to conduct focus group interviews as soon as possible after a sufficient number of patients have completed a full year of treatment with the CC-TRS. The focus group interviews will preferably take place with 6 to 12 informal caregivers, with a minimum of 4 people. In these focus group interviews, a topic list will be used, with topics referring to the overall experiences of the informal caregivers with the CC-TRS, its various components, and aspects of collaboration with the CC team. In the interviews, informal caregivers will also be asked to reflect on the perceived effects of the CC-TRS on the patients. The underlying principles of the CC-TRS will be discussed in conjunction with the specific features of CC-TRS that contribute to positive treatment outcomes. All interviews will be audiotaped and transcribed verbatim.

#### Care Professionals

To compare team characteristics, we will describe the standard of care provided by the participating teams as well as the composition of staff before the start of the intervention period. To measure experiences with the training program, care professionals will complete an evaluation form after completing the training.

During the implementation of the CC-TRS, 90-minute feedback meetings will be held every 3 months with all care professionals in the team as well as the team manager. During these meetings, concrete information about the implementation achievements of the team will be discussed to promote the implementation and execution of the CC-TRS. In addition, factors that either promoted or hindered the implementation of the CC-TRS at the patient level will be discussed in an endeavor to both identify practical solutions to the barriers experienced and learn from each other by sharing successful applications of the CC-TRS. All these feedback meetings will be audiotaped and summarized, with notable quotes being transcribed verbatim.

For each patient, there will be a group interview with involved care professionals at T12. Within this 1-hour group interview, the individual case will be discussed in detail with care professionals. A quantitative measure will form the input for this interview. In addition, a topic list will be drawn to discuss the process of implementation of the CC-TRS within that specific case and to explore associations between the implementation process and patient outcomes. All interviews will be audiotaped and transcribed verbatim.

The quantitative data will be entered into SPSS Statistics (version 26; IBM Corp). The transcribed texts from the qualitative interviews and feedback meetings will be entered into MAXQDA (version 2020).

### Analyses

We will perform a combination of qualitative and quantitative analyses in accordance with the method described by Stake [45]. In this multiple case study, the analysis of data will occur at 2 different levels: the individual case level (single-case analysis) and group level (cross-case analysis).

In the single-case analysis, data from different sources and perspectives will be analyzed for each individual case. On the basis of the document analysis; quantitative measurements; and interviews with patients, informal caregivers, and care professionals, we will draw up a comprehensive description of each case in order to gain insight into how the CC-TRS was offered to each individual patient, how this was experienced by different people (patients, informal caregivers, and professionals), the course of the treatment over time, and individual patient’ outcomes over time. For each case, we will examine which factors (CC-TRS-related and other) contribute to both positive and negative treatment outcomes. By integrating data at the case level, we can examine the factors that influence the effective application of the CC-TRS at the following levels: (1) treatment-related variables, (2) patient variables, (3) caregiver variables, (4) informal caregiver variables, (5) interactional variables, (6) organizational variables, and (7) other contextual variables. The single-case analysis will result in a detailed description of the relevant case, including tables with a summary of the findings in relation to our aforementioned research questions.

In the cross-case analyses, the findings from the 20 single-case analyses will be analyzed at the group level. In 3 ways, we will investigate the extent to which the implementation plans were successfully carried out. First, we will conduct an intervention check through document analysis. In this way, we systematically identify if and to what extent the interventions have been implemented. Second, we will discuss, through feedback meetings with team members, which components of the CC-TRS are implemented as planned and which components are not, whether all preconditions are met sufficiently, and discuss the teams’ needs to overcome experienced barriers regarding implementation. Feedback meetings will be audiotaped and summarized, with notable quotes being transcribed verbatim. Third, in supervision meetings, CNS and CC nurses will discuss their own considerations and explanations for the extent and manner in which interventions are implemented at the patient level. Noteworthy findings and considerations will be noted. We will quantitatively analyze the overall effects of CC-TRS on different outcome measures. To this end, exploratory analyses will be performed on patients (n=20) using the Friedman Test (nonparametric variant of the repeated measures analysis of variance). Next, we will descriptively analyze the quantitative data related to patients’ satisfaction with the CC-TRS program, by using the *Consumer Quality Index* and the questionnaire *Experiences with CC-TRS*. Following this, an overall analysis of individual interviews with patients will be conducted. Descriptive qualitative analyses will then be performed to explore the experiences of patients in the CC-TRS intervention program. Subsequently, the data from informal caregivers will be analyzed, first quantitatively (FPCR and the questionnaire *Evaluation informal caregivers CC-TRS*), followed by a qualitative descriptive analysis of the data generated in the focus group interviews. The next step will be to analyze the data pertaining to care professionals. The first quantitative analysis will be conducted on caregivers’ satisfaction with the training program. This will be followed by a qualitative analysis of the data generated through feedback meetings. Finally, data from the caregivers’ interviews will be descriptively analyzed. Through recourse to the method of content analysis [70], patient records will be analyzed to investigate the implementation of the CC-TRS.

The final step in the cross-case analysis will involve the integration of both qualitative and quantitative findings through the use of summary descriptions and tables drawn up in previous stages of the data analysis. In addition, the experiences of the patients, informal caregivers, and professionals will be presented at the group level. On the basis of our analyses, we will be able to describe the extent to which the CC-TRS was implemented and the factors determining its successful implementation. At both the individual and group levels, we will provide insight into the preliminary effects of the intervention, including the factors that contributed to these effects.

### Ethics Approval

The protocol was approved by the scientific committee of Reinier van Arkel (ID CWO1803) and the Medical Ethical Committee of the Vrije Universiteit University Medical Centre (ID A2019.475, 2018.071 and NL number NL64469.029.18). The procedures in this study are in accordance with the ethical standards of the responsible committee on human experimentation (institutional and national) and with the Declaration of Helsinki (1975) as revised in 2013 [71].

## Results

Recruitment commenced in October 2020, with the aim of enrolling 20 patients over 2 years. Data collection will be completed by the end of 2023, and the results will be published once all data are available for reporting.

## Discussion

### Strengths and Limitations

A strength of this study is, as undertreatment of TRS is a common problem [1,11,12], extensive attention was paid to the recruitment procedure by screening all patients every 3 months supervised by the quality consultants to evaluate if they are eligible for participation in the study. Therefore, we expect it to be feasible to include 20 patients within 3 teams in an inclusion period of 2 years.

There are several strengths in the study regarding the factors that are crucial for effective implementation [72]. This study will consider both the facilitating factors and barriers to implementation. For example, the CC-TRS is a bottom-up initiative carried out by the CNS in clinical practice. This is expected to enhance support among other care professionals in the implementation of the CC program. Both the board and management support the implementation of the CC-TRS, which creates favorable conditions in terms of financial support, commitment, and the availability of staff. Moreover, the CC-TRS includes an extensive training program for care professionals. To build support among care professionals and secure the division of tasks, a tailored implementation plan will be drawn up for each team in close collaboration with team members. Quality managers will be appointed to support the implementation process. Feedback meetings will be organized regularly to discuss and streamline the implementation process. We expect the implementation of the CC-TRS to be promoted because of this comprehensive strategy.

A special feature of the CC-TRS intervention and the multiple case study is that they are initiated and developed by mental health nurses. In the CC-TRS, the CNS will be the supervising practitioner and CC nurses will carry out the CC-TRS in close cooperation with the other members of the multidisciplinary treatment team. The PhD candidate is a CNS, and mental health nurses will perform measurements in the role of researchers. Thus, the CC-TRS might also have a positive effect on the professionalization of the nursing profession as a whole.

This study had some limitations. The first limitation is that this study has faced delays in execution owing to suboptimal conditions in the teams that needed to be resolved before the start of the study, such as the completion of other projects that required significant time from care professionals and the availability of a CNS on the team. In addition, the outbreak of the COVID-19 pandemic has caused a delay. As a result, there will be a considerable amount of time between the training of professionals and the start of the intervention. To solve this problem, we will provide a refresher course before the inclusion of patients. A second limitation of this study concerns the establishment of treatment adherence and treatment resistance. It is important to know whether a patient is taking antipsychotics. We assess medication adherence as best as possible in a way that we considered appropriate for our pragmatic study, namely by using data from the electronic prescription system in patients’ files and reports. In clinical practice, it is difficult to quantify the actual intake of medications. Our participants belong to an outpatient population, which means that it is not easy to reliably observe the intake of medication or count pills. With our inclusion criteria, we aimed to align with the consensus criteria for assessment and definition of TRS as described by the Treatment Response and Resistance in Psychosis Working Group [60]. A final limitation is that the self-developed questionnaires, that is, *Questionnaire Experiences with Treatment* and *Evaluation informal caregivers CC-TRS* are not validated and pilot-tested. For interested readers, the questionnaires are available in Dutch by contacting the first author.

Several other issues were carefully considered in this study. First, we consider the inclusion strategy of participants. In clinical practice, and also noticed by other researchers [73], besides the Diagnostic and Statistical Manual of Mental Disorders diagnoses of schizophrenia or schizoaffective disorder, other schizophrenia spectrum disorders are also diagnosed in cases of persistent psychotic symptoms. To avoid the risk of excluding patients who meet the criteria for TRS, we will include a broader range of Diagnostic and Statistical Manual of Mental Disorders diagnoses. At T0, we will determine whether a patient is eligible for inclusion in the study. The second consideration is the exclusion of inpatient treatment settings. We expect the CC-TRS to be important in inpatient long-term care units given the presence of (undertreated) patients with TRS in such units. However, for practical reasons and to limit the scope of our study, we now focus exclusively on outpatient settings. To determine the effectiveness of the intervention in both outpatient and inpatient treatment settings unambiguously, it is desirable to conduct an experimental study on the effects of the intervention. If our study shows that the CC-TRS has positive effects on the outpatient population, future research should determine whether these effects can also be achieved in the inpatient population.

### Future Directions

One of the goals of our study is improved recovery (symptomatic, functional, and personal) by offering integrated treatment opportunities for patients with TRS. It also addresses treatment pessimism among mental health professionals regarding our target group of patients. On the basis of the results of this study, we will be able to formulate recommendations for clinical practice regarding preconditions and factors that influence the optimization of treatment of patients with TRS.

### Conclusions

Although the interventions in the CC-TRS are all established evidence-based interventions, they have hitherto not been consistently delivered to patients using the framework of the CC model. We expect this study to gain insight into the process of implementation, preliminary outcomes, and experiences of care professionals, patients, and informal caregivers with the CC-TRS and to contribute to the optimization of treatment for patients with TRS.

## Abbreviations

CC: Collaborative Care
CC-TRS: Collaborative Care program for patients with treatment-resistant schizophrenia
CNS: clinical nurse specialist
EIT: Early Psychosis Intervention Team
FACT: Flexible Assertive Community treatment
FMI: Family Motivational Intervention
FPCR: Family Perceptions of Caregiving Role
SDM: shared decision-making
TRS: treatment-resistant schizophrenia
